# The *mgtCBR* mRNA Leader Secures Growth of *Salmonella* in Both Host and Non-host Environments

**DOI:** 10.3389/fmicb.2019.02831

**Published:** 2019-12-06

**Authors:** Myungseo Park, Hyunkeun Kim, Daesil Nam, Dae-Hyuk Kweon, Dongwoo Shin

**Affiliations:** ^1^Department of Integrative Biotechnology, College of Biotechnology and Bioengineering, Sungkyunkwan University, Suwon, South Korea; ^2^Department of Molecular Cell Biology, Sungkyunkwan University School of Medicine, Suwon, South Korea; ^3^Samsung Medical Center, School of Medicine, Sungkyunkwan University, Suwon, South Korea

**Keywords:** mRNA leader, MgtC, MgtB, ATP, *Salmonella*

## Abstract

Upon intracellular cues, bacterial mRNA leaders often form secondary structures that determine expression of a downstream protein-coding region(s), thereby providing bacteria with a mechanism to control the amounts of necessary proteins in the right locales. Here we describe a polycistronic mRNA leader that secures bacterial growth by preventing dysregulated expression of the protein-coding regions. In *Salmonella*, the *mgtCBR* mRNA encodes the virulence protein MgtC and the Mg^2+^ transporter MgtB. A mutant designed to produce leaderless *mgtCBR* mRNA induced MgtC and MgtB in conditions that promote *mgtC* transcription. The dysregulated expression of MgtC and MgtB impaired bacterial growth under all such non-host environments. While MgtC, but not MgtB, normally reduces ATP levels in a process requiring the F_1_F_0_ ATP synthase, dysregulated MgtC and MgtB reduced ATP levels independently of the F_1_F_0_ ATP synthase, which correlated with the mutant’s growth defect. The mutant showed dysregulated MgtC expression and attenuated survival inside macrophages. While MgtB normally does not affect the phenotype, MgtB impaired intramacrophage survival of the mutant in the presence of MgtC. We provide an example showing that a polycistronic mRNA leader prevents the dysregulated function of protein-coding regions to allow bacteria to proliferate across complex niches.

## Introduction

The bacterium *Salmonella enterica* serovar Typhimurium (hereafter referred to as *Salmonella*) is a facultative intracellular pathogen that can proliferate both inside and outside host cells. To survive under both conditions, *Salmonella* must tightly control the amounts of necessary proteins in each locale.

The PhoP/PhoQ two-component system in *Salmonella* consists of the response regulator PhoP and the sensor kinase PhoQ (Groisman, 2001). The PhoQ protein senses multiple signals, such as low (i.e., micromolar concentrations) Mg^2+^, acidic pH, and certain antimicrobial peptides, and phosphorylates the PhoP protein, rendering it functional as a transcriptional regulator (Garcia Vescovi et al., 1996; Bader et al., 2005; Prost et al., 2007). The gene expression programs regulated by PhoP/PhoQ enable *Salmonella* to adapt to low Mg^2+^ (Garcia Vescovi et al., 1996), survive in acidic pH (Foster and Hall, 1990), and acquire resistance against antimicrobial peptides (Fields et al., 1989). Moreover, activation of PhoP/PhoQ is a key event enabling *Salmonella* to survive inside the macrophage phagosome (Fields et al., 1989), which contains antimicrobial factors, including acidic pH and antimicrobial peptides.

The virulence protein MgtC contributes to the growth of *Salmonella* inside macrophages as well as in low Mg^2+^ conditions (Blanc-Potard and Groisman, 1997; Rang et al., 2007). These dual roles of MgtC are associated with its ability to reduce cytoplasmic ATP levels by directly acting on the bacterium’s own F_1_F_0_ ATP synthase (Lee et al., 2013; Pontes et al., 2015). MgtC also prevents hyperpolarization of *Salmonella* membrane (Lee et al., 2013). Given that MgtC plays this role independently of the F_1_F_0_ ATP synthase, this result suggests that MgtC could affect ATP levels by acting on a protein(s) other than the F_1_F_0_ ATP synthase (Lee et al., 2013).

**FIGURE 1:**
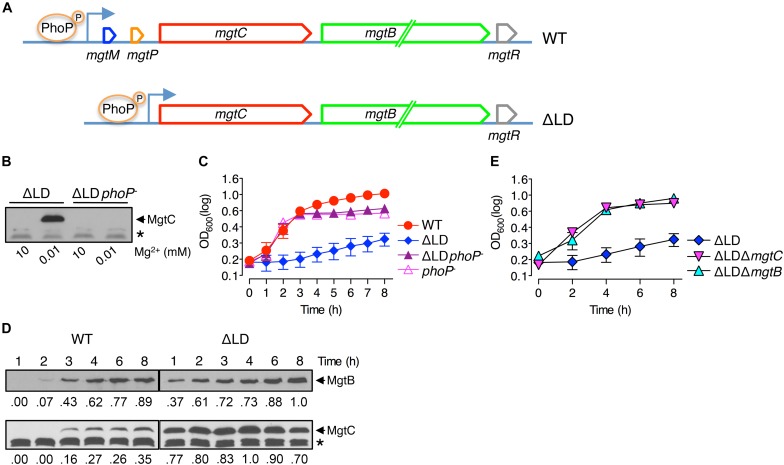
Dysregulated induction of MgtC and MgtB impairs growth of *Salmonella* in low Mg^2+^. **(A)** Schematic of the *mgtCBR* operon in the wild-type *Salmonella* (WT, 14028s) and ΔLD mutant (DN557). **(B)** Immunoblot analysis using anti-MgtC antibodies of crude extracts prepared from ΔLD and ΔLD *phoP*^–^ (DN558) strains. Bacteria were grown in N-minimal medium with 10 mM or 10 μM Mg^2+^ at pH 7.5 for 4 h. **(C,E)** Growth curves of wild-type (WT), ΔLD, ΔLD *phoP*^–^, *phoP*^–^ (MS7953s), ΔLD Δ*mgtC* (HK111), and ΔLD Δ*mgtB* (DN581) strains. Bacteria were grown in N-minimal medium with 10 μM Mg^2+^ at pH 7.5, and OD_600_ values were determined at the indicated time points. Means and standard deviations from three independent experiments are shown. **(D)** Immunoblot analysis using anti-MgtC and anti-MgtB antibodies of crude extracts prepared from wild-type (WT) and ΔLD strains grown in N-minimal medium with 10 μM Mg^2+^ at pH 7.5 and harvested at the indicated time points. The band indicated with an asterisk (^∗^) corresponds to a protein displaying cross-reactivity against the anti-MgtC antibody and serves as an internal loading control. Numbers below the blots correspond to relative levels of MgtC and MgtB at a given time point.

The MgtC protein, together with the Mg^2+^ transporter MgtB (Snavely et al., 1991b) and the regulatory peptide MgtR (Alix and Blanc-Potard, 2008), are produced from the *mgtCBR* operon, which is regulated at multiple levels (Lee and Lee, 2015). When *Salmonella* is placed in low Mg^2+^ or in acidic pH environments, activated PhoP directly promotes *mgtC* transcription (Shin and Groisman, 2005; Choi and Groisman, 2016). However, this event alone does not ensure production of full length *mgtCBR* mRNA. The *mgtCBR* mRNA contains a 296 nucleotides long leader that harbors two short open reading frames (ORFs), *mgtM* and *mgtP* (Figure 1A and Supplementary Figure S1). In response to intracellular levels of ATP or an amino acid, these ORFs can promote formation of alternative structures of the *mgtCBR* mRNA leader that determine the degree of *mgtC* expression (Lee and Groisman, 2012a, b; Lee et al., 2014). For instance, an increase in ATP levels and a decrease in proline levels in the cytoplasm affects the coupling/uncoupling of transcription of the *mgtCBR* mRNA leader with translation of *mgtM* and *mgtP*, respectively, to induce structures of the leader that allow transcription elongation into the *mgtC*-coding region (Lee and Groisman, 2012a, b; Lee et al., 2014). Such *mgtM*- and *mgtP* regulation enables *Salmonella* to induce *mgtC* transcription at levels that ensure its survival inside macrophages (Lee and Groisman, 2012a, b; Lee et al., 2014).

MgtC expression is also negatively controlled at the posttranscriptional level. In low Mg^2+^ conditions, PhoP binds to and activates the *amgR* promoter located in the *mgtC*–*mgtB* intergenic region (Lee and Groisman, 2010). This event produces the AmgR antisense RNA that promotes degradation of the *mgtC* mRNA in a process requiring the RNase E (Lee and Groisman, 2010). Inactivation of *amgR* transcription increases MgtC production and renders *Salmonella* more virulent in mice (Lee and Groisman, 2010). The regulatory peptide MgtR encoded by the *mgtCBR* operon binds to the MgtC protein and promotes MgtC degradation in a process dependent on the FtsH protease (Alix and Blanc-Potard, 2008). Although the *mgtR* mutant produces MgtC at much higher levels than the wild-type (WT) strain, the mutant is slightly attenuated for survival inside macrophages (Alix and Blanc-Potard, 2008).

In the present study, we designed a *Salmonella* mutant that produced leaderless *mgtCBR* mRNA. We found that in both host and non-host environments where the PhoP regulator is activated, removal of the *mgtCBR* mRNA leader caused *Salmonella* to induce MgtC and MgtB in a dysregulated manner, which impaired bacterial growth in such conditions. The dysregulated expression of MgtC and MgtB reduces ATP to abnormal levels in a process that does not require the F_1_F_0_ ATP synthase, and this reduction in ATP levels seems to be a cause for the *Salmonella* growth defect seen. Our study reveals that the role of the *mgtCBR* mRNA leader to prevent dysregulated expression of MgtC and MgtB is important for the growth of *Salmonella* both inside and outside host cells.

## Materials and Methods

### Bacterial Strains, Plasmids, and Growth Conditions

Bacterial strains and plasmids used in this study are listed in Table 1. All *S. enterica* serovar Typhimurium strains were derived from strain 14028s. Phage P22-mediated transductions were performed as described (Davis et al., 1980). Bacteria were grown at 37°C in N-minimal medium (Snavely et al., 1991a), pH 7.5 or pH 5.5, supplemented with 0.1% Casamino Acids, 38 mM glycerol, and the indicated concentrations of MgCl_2_. Ampicillin, kanamycin, and C18G peptide (AnaSpec) were used at 50, 30, and 5 μg/ml, respectively. For induction of genes from plasmids, isopropyl 1-thio-β-D-galatoside (IPTG) was used at the indicated concentrations.

**TABLE 1:** Bacterial strains and plasmids used in this study.

**Strain or plasmid**	**Genotype or relevant characteristics**	**References**
***S. enterica* serovar Typhimurium**		
14028s	Wild-type	Fields et al. (1986)
MS7953s	14028s *phoP7953*:Tn10	Fields et al. (1986)
DN552	14028s Δ*mgtR*	Choi E. et al. (2012)
DN557	14028s Δ*mgtCBR* mRNA leader (ΔLD)	This study
DN558	14028s ΔLD *phoP7953*:Tn10	This study
DN575	14028s ΔLD Δ*atpB*:Km^R^	This study
DN581	14028s ΔLD Δ*mgtB*	This study
DN582	14028s ΔLD Δ*mgtR*	This study
DN608	14028s ΔLD Δ*mgtCB*	This study
DN612	14028s *mgtM*(UAG) Δ*mgtB*	This study
DN649	14028s *mgtM*(UAG) Δ*mgtC*	This study
DN652	14028s *mgtM*(UAG) Δ*mgtCB*	This study
EG19307	14028s *mgtM*(UAG)	Lee and Groisman (2012a)
EN397	14028s Δ*mgtC*	Park et al. (2018)
EN481	14028s Δ*mgtB*	Park et al. (2018)
HK111	14028s ΔLD Δ*mgtC*	This study
HK468	14028s Δ*atpB*:Km^R^	This study
MS575	14028s ΔLD Δ*mgtC* Δ*atpB*:Km^R^	This study
MS576	14028s ΔLD Δ*mgtB* Δ*atpB*:Km^R^	This study
DN686	14028s ΔLD Δ*mgtCB* Δ*atpB*:Km^R^	This study
**Plasmids**		
pUHE21-2*lacI*^q^	P*_*lac*_* rep_*pMBI*_ Ap^R^ *lacI*^q^	Soncini et al. (1995)
p*mgtC*(ORF)	pUHE21-2*lacI^q^ mgtC* ORF	This study
pKD4	repR_6K γ_ Ap^R^FRT Km^R^ FRT	Datsenko and Wanner (2000)
pKD46	rep_*pSC101*_^*ts*^ Ap^R^ P*_araBAD_* γβ exo	Datsenko and Wanner (2000)
pCP20	rep_*pSC101*_^*ts*^ Ap^R^ Cm^R^ *cI857* λP_*R*_*flp*	Datsenko and Wanner (2000)

### Construction of Chromosomal Mutants and Plasmid

*Salmonella enterica* strains carrying a chromosomal gene deletion were constructed using the one-step gene inactivation method (Datsenko and Wanner, 2000) with necessary modifications. DN557 is a strain, in which the sequences encoding the *mgtCBR* mRNA leader regions were deleted from the chromosome. To construct DN557, the *tetRA* fragment was amplified from strain MS7953s using the primer pair Del701/Del702 and integrated into the chromosome of WT strain 14028s harboring the pKD46 plasmid (Datsenko and Wanner, 2000). The resulting strain HK514 keeps the *tetRA* genes in the *mgtCBR* mRNA leader-encoding region and pKD46 at 30°C. To obtain the engineered DNA fragment that is deleted for *mgtCBR* mRNA leader-encoding regions, the left and right arms of the DNA fragment were amplified from strain 14028s using primer pairs Del703/Del704 and Del705/Del706, respectively. Then, the second step of PCR was conducted on the mixture of the first step of PCR products using the primer pair Del703/Del706. The resulting PCR product was used to electroporate the HK514 strain, and the bacterial suspension was plated on medium containing fusaric acid and incubated at 42°C to select against the *tetRA* genes (Maloy and Nunn, 1981).

Strain HK111, in which the sequences corresponding to *mgtCBR* mRNA leader and *mgtC*-coding regions were removed from the chromosome, was similarly constructed. In the first step of PCR, two DNA fragments were amplified from strain 14028s using primer pairs Del703/Del704 and Del705/Del708. The second step of PCR was conducted on the mixture of the first step of PCR products using the primer pair Del703/Del708. The resulting PCR product was introduced into the strain HK514, and tetracycline-sensitive colonies were selected. Deletion of the sequences in the strains DN557 and HK111 was verified by nucleotide sequencing.

To delete the *mgtB* gene in the DN557 and HK111 strains, the kanamycin resistance gene (Km^R^) cassette from the pKD4 plasmid (Datsenko and Wanner, 2000) was amplified using the primer pair Del711/Del712. The resulting PCR product was used to electroporate the DN557 and HK111 strains carrying pKD46. To delete the *mgtC*, *mgtB*, and *mgtCB* genes in strain EG19307, the Km^R^ cassette from plasmid pKD4 was amplified using primer pairs Del713/Del714, Del711/Del712, and Del713/Del712, respectively. The respective PCR products were used to electroporate the EG19307 strain carrying pKD46. To delete the *mgtR* gene in strain DN557, the Km^R^ cassette from plasmid pKD4 was amplified using the primer pairs Del715/Del716. The respective PCR products were used to electroporate the DN557 strain carrying pKD46. The Km^R^ cassette was removed using plasmid pCP20 (Datsenko and Wanner, 2000). To delete the *atpB* gene in the 14028s strain, the Km^R^ cassette from plasmid pKD4 was amplified using the primer pair Del717/Del718, and the PCR product was used to electroporate the 14028s strain carrying pKD46. Deletion of the corresponding genes was verified by colony PCR. The sequences of all primers used for strain construction are listed in Supplementary Table S1.

Plasmid p*mgtC*(ORF) expresses the *mgtC* ORF from the *lac* promoter. The *mgtC* ORF was amplified using the primer pair Ex301/Ex302 and chromosomal DNA from the 14028s strain, and the *mgtC* ORF was then introduced between the *Eco*RI and *Pst*I restriction sites of pUHE21-2*lacI*^q^ (Soncini et al., 1995). Recombinant plasmid sequences were confirmed by nucleotide sequencing. The sequences of primers used for plasmid construction are listed in Supplementary Table S1.

### Immunoblot Analysis

Bacteria were grown in N-minimal medium for the indicated amounts of time. Equivalent amounts of bacterial cells normalized by OD_600_ values were collected, washed with phosphate-buffered saline (PBS), suspended in 0.15 ml SDS sample buffer (Laemmli sample buffer), and boiled. Whole-cell lysates were resolved on 12% SDS polyacrylamide gels, transferred to nitrocellulose membranes, and analyzed by immunoblot using anti-MgtC (Park et al., 2018), anti-MgtB (Choi et al., 2017), or anti-RpoA (NeoClone) antibodies. Membranes were incubated with anti-rabbit IgG horseradish peroxidase-linked antibodies (GE Healthcare), and bands were visualized using the ECL detection system (GE Healthcare). Protein levels were quantified using the ImageJ program (NIH).

### RNA Isolation and Quantitative Real-Time RT-PCR (qRT-PCR) Analysis

Bacteria were grown in N-minimal medium for the indicated amounts of time. The culture (0.5 ml) was removed and mixed with 1 ml RNAprotect Bacteria Reagent (Qiagen). RNA was isolated using the RNeasy Mini Kit (Qiagen) and treated with RNase-free DNase (Ambion). cDNA was synthesized from 0.1 μg of template RNA using the PrimeScript RT reagent Kit (Takara) and random primers (Life Technologies). Amounts of cDNA were quantified by real-time PCR using the SYBR Green Realtime PCR Master Mix (Toyobo) with an ABI7300 Sequence Detection System (Applied Biosystems). The following primer pairs were used for the detection of cDNA corresponding to *mgtC*, *mgtB*, and *gyrB* mRNAs: Q-mgtC-F/Q-mgtC-R, Q-mgtB-F/Q-mgtB-R, and Q-gyrB-F/Q-gyrB-R, respectively. Transcription levels of each gene were calculated from a standard curve obtained by PCR with the same primers and serially diluted genomic DNA. mRNA levels of target genes were normalized to *gyrB* mRNA levels. The sequences of primers are listed in Supplementary Table S1.

### Measurement of Intracellular ATP Levels

ATP levels were determined as previously described (Lee et al., 2013). Bacteria were grown in N-minimal medium for 4 h. Equivalent amounts of bacterial cells (0.5 × OD_600_) were removed, washed with PBS, and suspended in 0.5 ml of PBS. Nucleic acids were extracted by adding 100 ml of ice-cold 3 M perchloric acid. After incubation for 5 min, extracts were neutralized with 225 ml of neutralization buffer (1 M KOH, 0.5 M Tris, 0.5 M KCl) and centrifuged at 13,000 rpm and 25°C for 10 min. Fifty microliters of the supernatant were diluted with 50 ml of L buffer (25 mM KCl, 50 mM MgSO_4_, 100 mM HEPES pH 7.4), and ATP levels were measured using an ATP Determination Kit (Life Technologies) according to the manufacturer’s instruction. Statistical analysis of the data was conducted using the GraphPad Prism program (version 5.0).

### Macrophage Survival Assay

The experiment was conducted as previously described (Choi Y. et al., 2012). J774A.1 macrophage cells were grown in Dulbecco modified Eagle medium (DMEM) (Life Technologies) supplemented with 10% fetal bovine serum (FBS) and 1% Antibiotic-Antimycotic (Life Technologies) at 37°C under 5% CO_2_. Prior to bacterial infection, a monolayer of 1 × 10^5^ J774A.1 cells was prepared in a 24-well tissue culture plate and incubated in DMEM with 10% FBS for 1 h. Bacteria were cultured in Luria-Bertani (LB) medium for 18 h with aeration and opsonized in 20% normal mouse serum for 25 min at 37°C. Opsonized bacteria were diluted in DMEM with 10% FBS and added to the cell monolayer at a multiplicity of infection (MOI) of 10. After 1 h of incubation, the wells were washed three times with pre-warmed PBS to remove extracellular bacteria and then incubated for another 1 h with the pre-warmed medium with 100 μg/ml gentamicin to kill extracellular bacteria. The wells were washed three times with PBS and incubated in pre-warmed medium with 10 μg/ml of gentamicin. At the desired time points, the wells were washed three times with PBS and treated with 1% Triton X-100 for 10 min. The suspension was diluted in PBS and plated on LB agar plates to enumerate colony-forming units.

### Determination of MgtC Levels Inside Macrophages

J774A.1 macrophage and bacterial cells were cultured as described above. A monolayer of 1 × 10^6^ J774A.1 cells was prepared in a 6-well tissue culture plate. Opsonized bacteria were added to the cell monolayer at a MOI of 10. The wells were then treated as described above to remove extracellular bacteria. At the desired time points, the wells were washed three times with PBS and treated with 1% Triton X-100 for 30 min. The cell lysis mixture was centrifuged at 13,000 rpm for 10 min, and the bacterial cell pellet was suspended in 0.15 ml SDS sample buffer and boiled. MgtC levels in bacterial cell lysates were analyzed by immunoblot as described above.

### Measurement of Bacterial Growth Using a Plate Reader

Bacteria were grown in N-minimal medium with 10 mM MgCl_2_ at pH 7.5 to saturation. One milliliter of bacterial cells were collected, washed twice with medium not supplemented with MgCl_2_, and diluted 1:100 into wells of a 24-well plate containing 1 ml of medium with 10 μM or 1 mM MgCl_2_ at pH 7.5 or pH 5.5. The C18G peptide was added to medium at 5 μg/ml. A plate was covered with a Breathe-Easy sealing membrane (Sigma) to prevent evaporation. By using an xMark^TM^ Microplate Spectrophotometer (Bio-Rad), bacteria were cultivated at 37°C with shaking, and the OD_600_ values were measured every 5 min up to 8 h.

## Results

### A *Salmonella* Strain Designed to Produce Leaderless *mgtCBR* mRNA Is Severely Impaired for Growth in Low Mg^2+^

We investigated the behaviors of *Salmonella* that produces leaderless *mgtCBR* mRNA. We deleted the sequences specifying the *mgtCBR* mRNA leader regions that participate in forming regulatory structures from the chromosome without leaving any heterologous sequences behind (Figure 1A and Supplementary Figure S1). The resulting strain (ΔLD) still possessed PhoP-dependent Mg^2+^ regulation of *mgtC* expression. The MgtC protein was detected in the ΔLD strain grown in defined medium supplemented with 10 μM Mg^2+^ but not in medium with 10 mM Mg^2+^ for 4 h, and the low Mg^2+^-induced MgtC production did not occur in the strain carrying a *phoP*^–^ allele (Figure 1B). (Note that the medium pH was adjusted to 7.5, unless otherwise stated.)

While preparing bacterial cultures, we repeatedly observed that the ΔLD strain did not grow well in low Mg^2+^. During experiments, *Salmonella* strains were initially grown to saturation in medium with 10 mM Mg^2+^ and then diluted to OD_600_ values of ∼0.15 in medium with 10 μM Mg^2+^. After a growth lag for the first 1 h, the WT strain grew logarithmically up to 3 h and then displayed slow linear growth for the subsequent 5 h (Figure 1C), generating a typical growth curve in 10 μM Mg^2+^ (Soncini et al., 1996; Blanc-Potard and Groisman, 1997; Park et al., 2018). However, the ΔLD strain did not achieve logarithmic growth and only grew in a slow linear manner during the entire 8 h (Figure 1C).

### The ΔLD Strain Expresses MgtC and MgtB in Low Mg^2+^ in a Dysregulated Manner

To understand the molecular basis underlying the growth defect seen in the ΔLD strain, we compared expression of *mgtC* and *mgtB* between the WT and ΔLD strains. When *Salmonella* is placed in low Mg^2+^, PhoP directly promotes transcription initiation of the *mgtC* gene (Shin and Groisman, 2005). However, *Salmonella* can produce MgtC protein at detectable levels after cytoplasmic Mg^2+^ levels drop to a certain threshold (Yeom et al., 2017; Park et al., 2018). Given that a secondary structure of the *mgtCBR* mRNA leader prevents transcription elongation from reaching the *mgtC*-coding region (Lee and Groisman, 2012a, b; Lee et al., 2014), we reasoned that the expression kinetics of *mgtC* and *mgtB* must be different between the WT and ΔLD strains.

When placed in medium with 10 μM Mg^2+^, the WT strain produced *mgtC* mRNA at very low levels for the first 2 h (Supplementary Figure S2A). The *mgtC* mRNA levels then increased at 3 h by ∼10-fold, and this high-level production continued for the following 5 h (Supplementary Figure S2A). By contrast, under the same growth condition, the ΔLD strain produced *mgtC* mRNA at levels similar to the maximum levels of the WT as early as at 1 h and maintained these levels for the following 7 h (Supplementary Figure S2A). Moreover, consistent with the notion that the *mgtC* and *mgtB* mRNAs constitute a polycistronic mRNA (Alix and Blanc-Potard, 2008; Lee and Groisman, 2010), the WT and ΔLD strains highly induced *mgtB* mRNA at the delayed (i.e., 3 h) and the early (i.e., 1 h) time points, respectively (Supplementary Figure S2B).

We also determined levels of the MgtC and MgtB proteins over time during bacterial growth in 10 μM Mg^2+^. Consistent with previous findings (Yeom et al., 2017; Park et al., 2018) and the *mgtC* mRNA levels (Supplementary Figure S2A), MgtC was detected in the WT strain only after 3 h (Figure 1D). In contrast, and as observed with *mgtC* mRNA production (Supplementary Figure S2A), the ΔLD strain produced detectable levels of MgtC as early as 1 h of growth (Figure 1D). Notably, despite the finding that the maximum levels of *mgtC* mRNA were comparable between the two strains (Supplementary Figure S2A), MgtC levels were ∼4-fold higher in the ΔLD strain than in the WT strain at 4 h (Figure 1D). As observed with *mgtB* mRNA production (Supplementary Figure S2B), the WT and ΔLD strains produced detectable levels of MgtB in parallel with MgtC (Figure 1D). However, in contrast to MgtC levels, MgtB levels detected after 4 h were similar between the two strains (Figure 1D).

In medium with 10 μM Mg^2+^, WT *Salmonella* grew logarithmically for 3 h by consuming Mg^2+^ in the medium (Figure 1C). During this time period, the ΔLD strain produced the Mg^2+^ transporter MgtB at much higher levels (Figure 1D) with even lower growth yields than the WT strain (Figure 1C), suggesting that cytoplasmic Mg^2+^ levels in the mutant are at least not lower than the WT levels. Taken together, these results suggest that removal of the leader from the *mgtCBR* mRNA causes *Salmonella* to induce MgtC and MgtB in a dysregulated manner even prior to its experiencing low cytoplasmic Mg^2+^ stress.

### MgtC and MgtB Impair Growth of the ΔLD Strain in Low Mg^2+^

As previously observed (Soncini et al., 1996; Blanc-Potard and Groisman, 1997), in medium with 10 μM Mg^2+^, the *phoP*^–^ strain grew logarithmically in a manner similar to the WT strain but showed defective growth in the slow-growth phase (Figure 1C). The ΔLD *phoP*^–^ strain, which failed to induce MgtC expression (Figure 1B), showed logarithmic growth, with a growth curve similar to that of the *phoP*^–^ strain (Figure 1C). This result suggests that the growth defect of the ΔLD strain may be associated with dysregulated MgtC induction. Consistent with this prediction, the ΔLD Δ*mgtC* strain recovered the ability of logarithmic growth (Figure 1E).

Because the ΔLD strain produced MgtB in a dysregulated manner as well as MgtC (Figure 1D), we next explored whether the *mgtB* gene was also responsible for the growth defect. Indeed, in medium with 10 μM Mg^2+^, *mgtB* deletion enabled the ΔLD strain to grow logarithmically (Figure 1E). In the ΔLD background, *mgtC* deletion did not affect MgtB production, and vice versa (Supplementary Figure S3), suggesting that the growth phenotypes of the ΔLD Δ*mgtC* and ΔLD Δ*mgtB* strains are due to lack of MgtC- and MgtB function, respectively.

The *mgtR* gene in the *mgtCBR* operon (Figure 1A) encodes the regulatory peptide MgtR that controls MgtC levels (Alix and Blanc-Potard, 2008). Because the ΔLD strain is also predicted to produce MgtR in a dysregulated manner, we further examined whether *mgtR* deletion affects growth of the ΔLD strain. However, the ΔLD Δ*mgtR* strain grew in a manner similar to the ΔLD strain (Supplementary Figure S4). Together these results indicate that when produced in a dysregulated manner, MgtC and MgtB impair the growth of *Salmonella* under low Mg^2+^ conditions.

### In the ΔLD Strain, MgtC and MgtB Contribute to ATP Reduction to Abnormal Levels in a Process That Does Not Require F_1_F_0_ ATP Synthase

**FIGURE 2:**
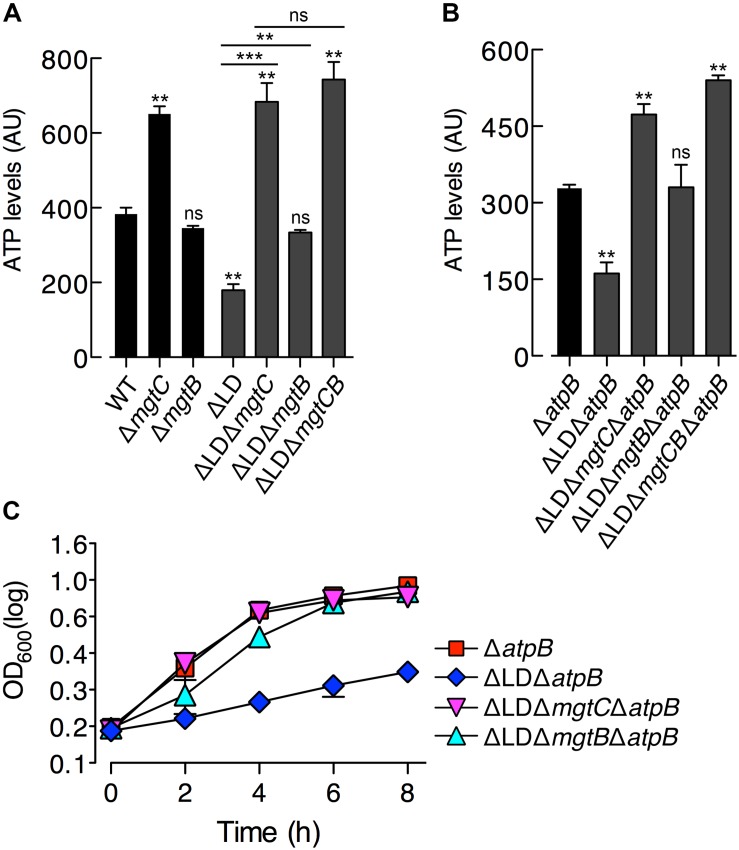
Dysregulated expression of MgtC and MgtB reduces ATP to abnormal levels in a process that does not require F_1_F_0_ ATP synthase. ATP levels were determined in wild-type (WT, 14028s), Δ*mgtC* (EN397), Δ*mgtB* (EN481), ΔLD (DN557), ΔLD Δ*mgtC* (HK111), ΔLD Δ*mgtB* (DN581), and ΔLD Δ*mgtCB* (DN608) strains **(A)** as well as in Δ*atpB* (HK468), ΔLD Δ*atpB* (DN575), ΔLD Δ*mgtC* Δ*atpB* (MS575), ΔLD Δ*mgtB* Δ*atpB* (MS576), and ΔLD Δ*mgtCB* Δ*atpB* (DN686) strains **(B)**. Bacteria were grown in N-minimal medium with 10 μM Mg^2+^ at pH 7.5 for 4 h. Data depicted in arbitrary units (AU) are means and standard deviations from three independent experiments. ^∗∗^*P* < 0.01, ^∗∗∗^*P* < 0.001, two-tailed *t*-test with each sample vs. WT **(A)** and with each sample vs. Δ*atpB* **(B)**, ns, not significant. **(C)** Growth curves of *Salmonella* strains, Δ*atpB* (HK468), ΔLD Δ*atpB* (DN575), ΔLD Δ*mgtC* Δ*atpB* (MS575), and ΔLD Δ*mgtB* Δ*atpB* (MS576). Bacteria were grown in N-minimal medium with 10 μM Mg^2+^ at pH 7.5, and OD_600_ values were determined at the indicated time points. Data are representative of three independent experiments, and means and standard deviations from three independent experiments.

Given that MgtC functions to reduce cytoplasmic ATP levels by acting on the F_1_F_0_ ATP synthase (Lee et al., 2013) and that the ΔLD strain produced higher levels of MgtC (Figure 1D), we reasoned that ATP levels may be different between the ΔLD and WT strains. We thus determined ATP levels in *Salmonella* at 4 h after growth in medium with 10 μM Mg^2+^, in which both WT and ΔLD strains produced MgtC at detectable levels (Figure 1D). Similar to previous finding (Lee et al., 2013), ATP levels were higher in the Δ*mgtC* strain than in the WT strain (Figure 2A). In contrast, the ΔLD strain exhibited ∼2-fold lower ATP levels than the WT (Figure 2A). The ATP reduction in the ΔLD strain was dependent on MgtC, as deleting *mgtC* increased the ATP levels by ∼3-fold (Figure 2A).

Despite the findings that MgtB does not impact ATP levels in the WT strain (Lee et al., 2013; Figure 2A) and that MgtB levels were comparable between the WT and ΔLD strains (Figure 1D), we examined if MgtB affected ATP levels in the ΔLD strain. Interestingly, deletion of the *mgtB* gene elevated ATP levels in the ΔLD strain by ∼2-fold (Figure 2A). Moreover, additional deletion of *mgtC* further increased ATP levels of the ΔLD Δ*mgtB* strain to levels of the ΔLD Δ*mgtC* strain (Figure 2A). Together these results suggest that in the ΔLD strain, MgtB might contribute to ATP reduction in a manner dependent on MgtC.

The MgtC protein inhibits ATP synthesis by directly binding to the F_0_ *a* subunit of the ATP synthase (Lee et al., 2013). Consistent with this observation, MgtC-dependent ATP reduction is no longer observed in the absence of the *atpB* gene, which encodes the F_0_ *a* subunit (Lee et al., 2013). To explore whether ATP reduction in the ΔLD strain requires the F_1_F_0_ ATP synthase, we determined ATP levels in a set of Δ*atpB* strains grown in 10 μM Mg^2+^ for 4 h. We found that ATP levels were still ∼2-fold lower in the ΔLD Δ*atpB* strain than in the Δ*atpB* strain (Figure 2B). Moreover, deleting *mgtC* and *mgtB* increased ATP levels in the ΔLD Δ*atpB* strain by ∼3- and ∼2-fold, respectively (Figure 2B). Additional deletion of *mgtC* further increased ATP levels of the ΔLD Δ*mgtB* Δ*atpB* strain to levels of the ΔLD Δ*mgtC* Δ*atpB* strain (Figure 2B). Together these results suggest that dysregulated expression of MgtC and MgtB reduces ATP to abnormally low levels in a process that does not require F_1_F_0_ ATP synthase.

### ATP Reduction Impairs Growth of the ΔLD Strain in Low Mg^2+^

In medium with 10 μM Mg^2+^, the ΔLD strain, which displayed lower ATP levels than the WT (Figure 2A), showed impaired growth (Figure 1C). In contrast, the ΔLD Δ*mgtC* strain, which displayed ATP levels as high as the Δ*mgtC* strain (Figure 2A), recovered growth to levels of the Δ*mgtC* strain (Supplementary Figure S5A). A similar relationship between ATP level and growth behavior was also observed between the ΔLD Δ*mgtB* and Δ*mgtB* strains (Figure 2A and Supplementary Figure S5B).

Growth behaviors of the strains carrying an *atpB* deletion also correlated with their ATP levels. In medium with 10 μM Mg^2+^, the Δ*atpB* strain grew in a manner similar to the WT strain (Figure 2C). However, the ΔLD Δ*atpB* strain, which exhibited lower ATP levels than the Δ*atpB* strain (Figure 2B), showed severely impaired growth (Figure 2C). Additionally, deleting *mgtC* and *mgtB*, which each increased ATP levels in the ΔLD Δ*atpB* strain (Figure 2B), recovered growth of the ΔLD Δ*atpB* strain (Figure 2C). Together these results suggest that the ATP reduction led by MgtC and MgtB independent of F_1_F_0_ ATP synthase caused impaired growth of the ΔLD strain in low Mg^2+^.

### Dysregulated Induction of MgtC and MgtB Impairs *Salmonella* in High Mg^2+^ Environments With Acidic pH or an Antimicrobial Peptide

**FIGURE 3:**
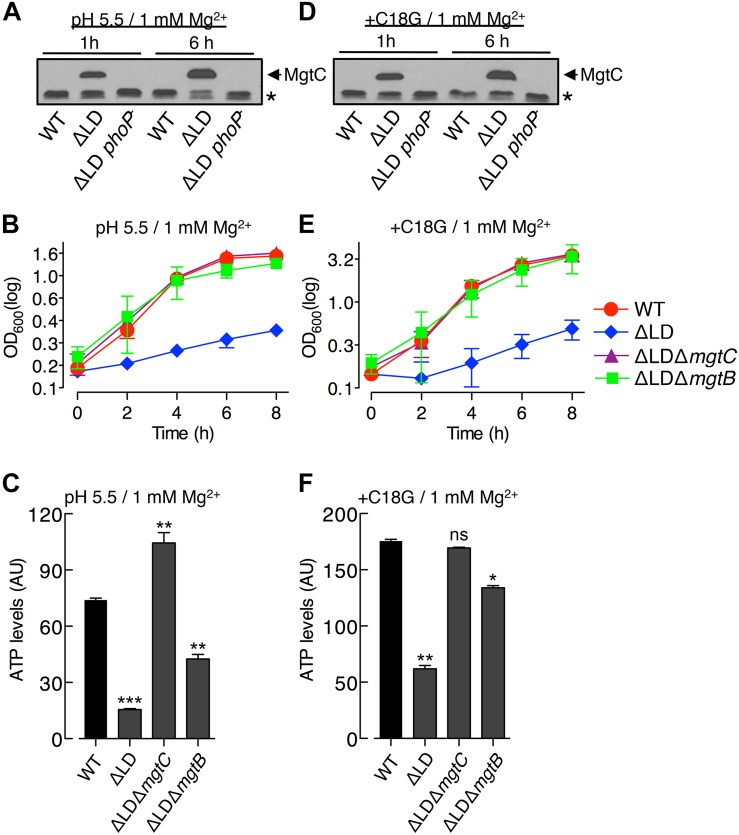
Dysregulated induction of MgtC and MgtB impairs growth of *Salmonella* in high Mg^2+^ with acidic pH or an antimicrobial peptide. Bacteria were grown in N-minimal medium with 1 mM Mg^2+^ at pH 5.5 **(A–C)** or with 1 mM Mg^2+^ and 5 μg/ml C18G peptide at pH 7.5 **(D–F)**. **(A,D)** Immunoblot analysis of MgtC levels in wild-type (WT), ΔLD (DN557), and ΔLD *phoP*^–^ (DN558) strains at 1 and 6 h after growth. The band indicated with an asterisk (^∗^) corresponds to a protein displaying cross-reactivity against the anti-MgtC antibody and serves as an internal loading control. **(B,E)** Growth curves of wild-type, ΔLD, ΔLD Δ*mgtC* (HK111), and ΔLD Δ*mgtB* (DN581) strains. OD_600_ values were determined at the indicated time points. Means and standard deviations from three independent experiments are shown. **(C,F)** ATP levels were determined in wild-type (WT), ΔLD, ΔLD Δ*mgtC*, and ΔLD Δ*mgtB* strains at 4 h after growth. Data depicted in arbitrary units (AU) are means and standard deviations from three independent experiments. ^∗^*P* < 0.05, ^∗∗^*P* < 0.01, ^∗∗∗^*P* < 0.001, two-tailed *t*-test with each sample vs. WT; ns, not significant.

The PhoP regulator can be activated even in high (i.e., millimolar concentrations) Mg^2+^ by mildly acid pH (Prost et al., 2007; Choi and Groisman, 2016) or certain antimicrobial peptides (Bader et al., 2005). Given that PhoP promotes *mgtC* transcription at acidic pH (Choi and Groisman, 2016), we speculated about the behavior of the ΔLD strain under such conditions. We first determined MgtC levels in *Salmonella* grown in medium supplemented with 1 mM Mg^2+^ adjusted to pH 5.5. The ΔLD strain produced detectable levels of MgtC at 1 and 6 h after growth in a PhoP-dependent manner (Figure 3A). However, in the WT strain, MgtC was not detected at either time point (Figure 3A).

In the acidified medium, the ΔLD strain grew in a linear, slow fashion during the entire 8 h, which was in contrast to the WT strain that grew logarithmically for the first 4 h and continued to grow slowly for the remaining 4 h (Figure 3B). The growth defect seen in the ΔLD strain was also dependent on MgtC and MgtB, as evidenced by the finding that deleting *mgtC* and *mgtB* restored growth to the ΔLD strain (Figure 3B). Growth behaviors of the strains correlated with their ATP levels. ATP levels were ∼4-fold lower in the ΔLD strain than in the WT strain (Figure 3C). Deleting *mgtC* and *mgtB* increased ATP levels in the ΔLD strain by ∼6- and ∼2.5-fold, respectively (Figure 3C).

We next examined growth in medium containing 1 mM Mg^2+^ and sublethal concentrations of C18G, an antimicrobial peptide that activates the PhoP regulator (Bader et al., 2005). Again, only the ΔLD strain induced detectable MgtC (Figure 3D) and displayed a growth defect dependent on MgtC and MgtB (Figure 3E). Moreover, the ΔLD strain displayed reduced ATP levels in a manner associated with MgtC and MgtB (Figure 3F). Together, these results indicate that even if *mgtC* transcription is activated in high Mg^2+^ by acidic pH or an antimicrobial peptide, the *mgtCBR* mRNA leader prevents dysregulated induction of MgtC and MgtB, which is necessary for *Salmonella* to normally grow in these environments.

### MgtC and MgtB Impair Growth of the ΔLD Strain Inside Macrophages in a Manner Not Associated With the F_1_F_0_ ATP Synthase

**FIGURE 4:**
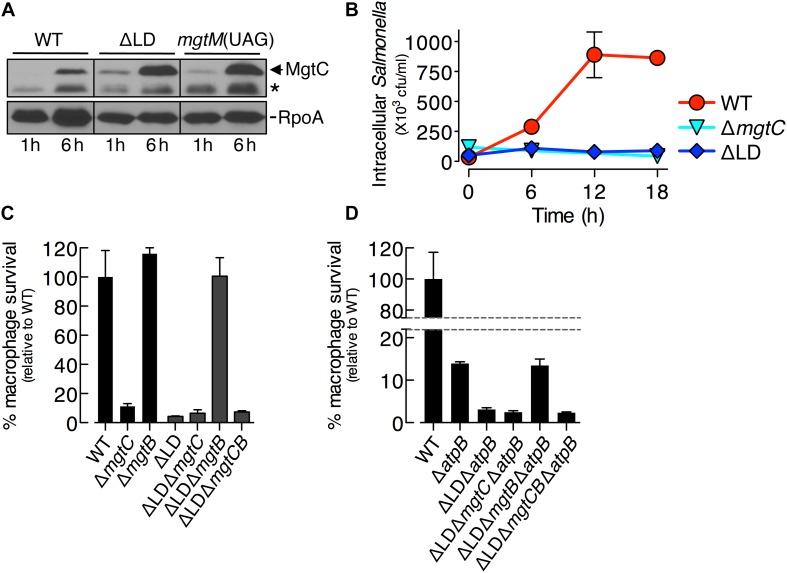
Dysregulated induction of MgtC and MgtB impairs *Salmonella* inside macrophages. **(A)** Immunoblot analysis of MgtC and RpoA determined in the wild-type (WT, 14028s), ΔLD (DN557), and *mgtM*(UAG) (EG19307) strains at 1 and 6 h after engulfment by J774A.1 macrophages. The band indicated with an asterisk (^∗^) corresponds to a protein displaying cross-reactivity against the anti-MgtC antibody. **(B)** Growth behaviors of wild-type, Δ*mgtC* (EN397), and ΔLD strains inside macrophages. After infection of macrophages, intracellular numbers of bacteria were determined at the indicated time points. **(C,D)** Intramacrophage survival of wild-type, Δ*mgtC*, Δ*mgtB* (EN481), ΔLD, ΔLD Δ*mgtC* (HK111), ΔLD Δ*mgtB* (DN581), ΔLD Δ*mgtCB* (DN608), Δ*atpB* (HK468), ΔLD Δ*atpB* (DN575), ΔLD Δ*mgtC* Δ*atpB* (MS575), ΔLD Δ*mgtB* Δ*atpB* (MS576), and ΔLD Δ*mgtCB* Δ*atpB* (DN686) strains. After infection of macrophages, the intracellular numbers of bacteria at 18 h were divided by those at 1 h. The percentage of survival of each mutant relative to the wild-type strain is presented. Means and standard deviations from three independent experiments are shown.

The notion that MgtC induction is necessary for *Salmonella* to survive inside macrophages (Blanc-Potard and Groisman, 1997) led us to explore behaviors of the ΔLD strain inside macrophages. We first determined MgtC levels in cell extracts prepared from *Salmonella* engulfed by macrophages. After entry into macrophages, the WT strain induced detectable MgtC at 6 h but not at 1 h, whereas the ΔLD strain induced MgtC at both time points (Figure 4A). This result indicates that the *mgtCBR* mRNA leader prevents earlier induction of MgtC during *Salmonella* infection of macrophages.

We then examined the growth of *Salmonella* inside macrophages. Intracellular numbers of WT *Salmonella* increased at 6 h after engulfment by ∼8-fold and peaked at 12 h by ∼25-fold, and these numbers were maintained at 18 h (Figure 4B). By contrast, during the entire experiment, the ΔLD strain was unable to grow inside macrophages, resembling the phenotype of the Δ*mgtC* strain (Figure 4B).

We also determined intramacrophage survival of *Salmonella* mutants relative to the WT strain at 18 h post infection. The ΔLD strain displayed only ∼5% survival compared with the WT strain (Figure 4C). The ΔLD Δ*mgtC* strain showed a low survival similar to the ΔLD strain, whereas the ΔLD Δ*mgtB* strain exhibited WT levels of survival (Figure 4C). These findings suggest that the phenotype of the ΔLD strain is dependent on MgtB and that MgtC alone supports survival of the ΔLD strain unless MgtB is present. Furthermore, the similarly low survival between the ΔLD Δ*mgtC* and the ΔLD Δ*mgtCB* strains (Figure 4C) excludes the possibility that MgtB alone might impair survival of the ΔLD strain in the absence of MgtC. Together these results suggest that, when induced inside macrophages in a dysregulated manner, MgtC and MgtB impair intramacrophage growth of *Salmonella*.

The role of MgtC in supporting survival of *Salmonella* inside macrophages is due to its ability to inhibit the F_1_F_0_ ATP synthase (Lee et al., 2013). Consistent with this notion, *mgtC* deletion did not further impair the Δ*atpB* strain, which showed much lower survival than the WT strain (Figure 4D; Lee et al., 2013). By contrast, the ΔLD Δ*atpB* strain displayed more attenuated survival than the Δ*atpB* strain (Figure 4D). The ΔLD Δ*mgtC* Δ*atpB* strain showed a low survival similar to the ΔLD Δ*atpB* strain, whereas survival of the ΔLD Δ*mgtB* Δ*atpB* strain increased to levels of the Δ*atpB* strain (Figure 4D). Intramacrophage survival was similarly low between the ΔLD Δ*mgtC* Δ*atpB* and the ΔLD Δ*mgtCB* Δ*atpB* strains (Figure 4D). These results suggest that MgtC and MgtB impair intramacrophage growth of the ΔLD strain in a manner not associated with F_1_F_0_ ATP synthase. Moreover, given that MgtC and MgtB reduced ATP to abnormal levels independently of the F_1_F_0_ ATP synthase in the ΔLD strain (Figure 2), this result suggests that such ATP reduction could also inhibit growth of *Salmonella* in host environments.

### A *Salmonella* Mutant Lacking *mgtM* Translation Displays Behaviors Resembling Those of the ΔLD Strain

The *mgtCBR* mRNA leader harbors the two short ORFs *mgtM* and *mgtP* (Figure 1A). Translation of *mgtM* and *mgtP* affects *mgtC* expression by affecting conformational changes in the leader (Lee and Groisman, 2012a, b; Lee et al., 2014). Thus, we hypothesized mutations in the leader that cause dysregulated induction of MgtC and MgtB might confer ΔLD strain-like phenotypes to *Salmonella*. We focused on the role of *mgtM*, as mutations preventing normal *mgtM* translation greatly increased β-galactosidase activity produced by *Salmonella* carrying a chromosomal *mgtC*–*lacZ* fusion (Lee and Groisman, 2012a).

**FIGURE 5:**
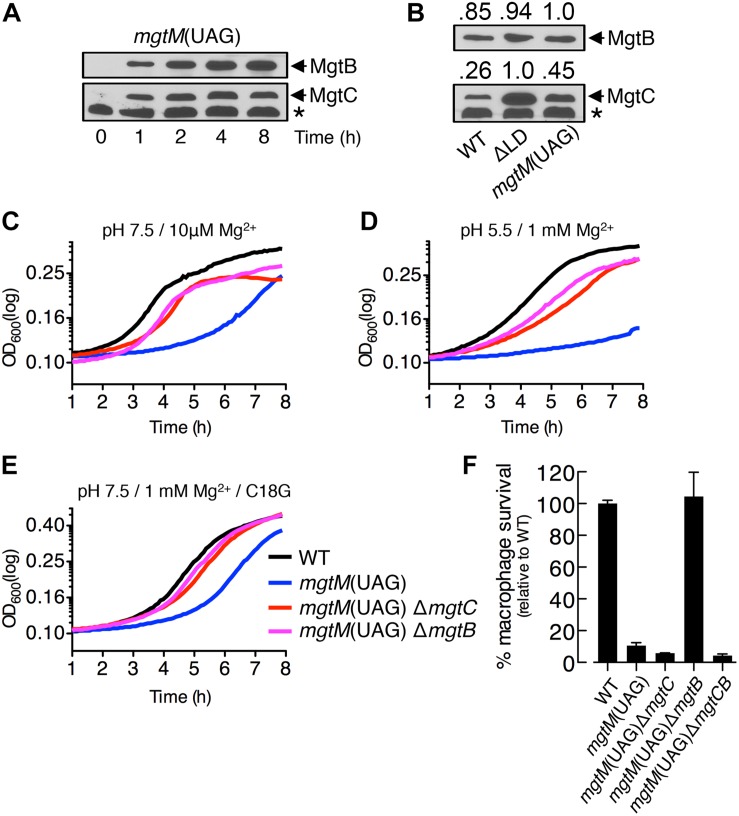
A conformation of the *mgtCBR* mRNA leader that causes dysregulated induction of MgtC and MgtB impairs *Salmonella* in host and non-host environments. **(A,B)** Immunoblot analysis of MgtC and MgtB in wild-type (WT, 14028s), ΔLD (DN557), and *mgtM*(UAG) (EG19307) strains. Bacteria were grown in N-minimal medium with 10 μM Mg^2+^ at pH 7.5 and harvested at the indicated time points **(A)** or at 4 h **(B)**. The band indicated with an asterisk (^∗^) corresponds to a protein displaying cross-reactivity against the anti-MgtC antibody. In panel **(B)**, numbers above the blots correspond to relative levels of MgtC and MgtB in each lane. **(C–E)** Growth curves of wild-type, *mgtM*(UAG) (EG19307), *mgtM*(UAG) Δ*mgtC* (DN649), and *mgtM*(UAG) Δ*mgtB* (DN612) strains. Bacteria were grown in N-minimal medium with 10 μM Mg^2+^ at pH 7.5 **(C)**, with 1 mM Mg^2+^ at pH 5.5 **(D)**, or with 1 mM Mg^2+^ and 5 μg/ml C18G peptide at pH 7.5 **(E)**, and OD_600_ values were determined every 5 min using a plate reader. Data are representative of three independent experiments, which gave similar results. **(F)** Intramacrophage survival of wild-type, *mgtM*(UAG), *mgtM*(UAG) Δ*mgtC*, *mgtM*(UAG) Δ*mgtB*, and *mgtM*(UAG) Δ*mgtCB* (DN652) strains was determined as described in the legend of Figure 4. Means and standard deviations from three independent experiments are shown.

We determined levels of MgtC and MgtB in the *mgtM*(UAG) strain, in which the start codon UUG of *mgtM* was replaced with the amber stop codon UAG (Lee and Groisman, 2012a). When the *mgtM*(UAG) strain was placed in medium with 10 μM Mg^2+^, both MgtC and MgtB were induced to detectable levels as early as at 1 h, and these levels were maintained for the following 7 h (Figure 5A), resembling the temporal production of MgtC and MgtB in the ΔLD strain (Figure 1D). Four hours after growth, the *mgtM*(UAG) strain produced MgtC at levels ∼2-fold higher than the WT strain and ∼2-fold lower than the ΔLD strain (Figure 5B). However, MgtB levels were similar among the three strains (Figure 5B).

We also found that the *mgtM*(UAG) strain exhibited growth defects in all host and non-host environments that promote *mgtC* transcription. When placed in medium with 10 μM Mg^2+^, the *mgtM*(UAG) strain entered logarithmic growth later and displayed lower growth yields during logarithmic growth than the WT strain (Figure 5C). However, deleting *mgtC* and *mgtB* corrected the logarithmic growth of the *mgtM*(UAG) strain (Figure 5C). The growth defect was also observed in medium with 1 mM Mg^2+^ and pH 5.5 as well as in medium with 1 mM Mg^2+^ and the C18G antimicrobial peptide (Figures 5D,E). Again, deletions of *mgtC* and *mgtB* restored growth of the *mgtM*(UAG) strain under these conditions (Figures 5D,E).

Survival of the *mgtM*(UAG) strain inside macrophages was only ∼10% relative to the WT strain (Figure 5F). While *mgtB* deletion increased survival of the *mgtM*(UAG) strain to WT levels, deletion of *mgtC* or both *mgtC* and *mgtB* slightly decreased survival of the strain to similar levels (Figure 5F). These results suggest that MgtC and MgtB impair growth of the *mgtM*(UAG) strain inside macrophages. Taken together, our results emphasize that the function of the *mgtCBR* mRNA leader in preventing dysregulated induction of MgtC and MgtB is necessary for *Salmonella* to proliferate in both host and non-host environments.

## Discussion

The *mgtCBR* mRNA leader, which contains two short ORFs for *mgtM* and *mgtP*, possesses tandem attenuators that sense two distinct signals (Lee and Groisman, 2012a, b; Lee et al., 2014). In response to acidic pH, an increase in ATP levels in the cytoplasm affects coupling/uncoupling of transcription of the leader with *mgtM* translation, inducing a conformation of the leader that promotes expression of the *mgtC*-coding region (Lee and Groisman, 2012a). Likewise, a decrease of intracellular proline promotes *mgtC* expression by affecting the events between leader transcription and *mgtP* translation (Lee and Groisman, 2012b; Lee et al., 2014).

In this study, we sought to further understand the physiological significance of the *mgtCBR* mRNA leader. We investigated two *Salmonella* mutants: the ΔLD strain, in which the sequences specifying the *mgtCBR* mRNA leader were removed (Figure 1A), and the *mgtM*(UAG) strain, in which the start codon of *mgtM* was replaced with a stop codon, resulting in the locking of the leader in a conformation allowing expression of the *mgtC*-coding region (Lee and Groisman, 2012a). Of note, MgtC expression was not constitutive but still inducible in these two strains; the ΔLD strain produced detectable levels of MgtC in 10 μM Mg^2+^ but not in 10 mM Mg^2+^ (Figure 1B), and MgtC was not detected in the *mgtM*(UAG) strain immediately at transfer from 10 mM to 10 μM Mg^2+^ (Figure 5A).

When *Salmonella* is placed in 10 μM Mg^2+^, the PhoP regulator promotes transcription initiation of *mgtC* (Shin and Groisman, 2005). However, this event alone does not ensure MgtC production. *Salmonella* logarithmically grows for the first few hours in 10 μM Mg^2+^ by consuming Mg^2+^ in the medium and then displays slow linear growth (Figure 1C; Soncini et al., 1996; Blanc-Potard and Groisman, 1997; Park et al., 2018). MgtC production is detectable after *Salmonella* enter into the slow-growth phase (Figures 1C,D; Yeom et al., 2017; Park et al., 2018). The decrease of cytoplasmic Mg^2+^ levels causes the onset of slow-growth phase (Pontes et al., 2016). The observation that MgtC induction is further delayed when *Salmonella* is grown in 50 μM Mg^2+^ (Park et al., 2018) further supports the relation between decreased cytoplasmic Mg^2+^ levels and MgtC induction. In contrast, both the ΔLD and *mgtM*(UAG) strains produced MgtC at detectable levels even during the time period corresponding to the logarithmic growth phase of the WT strain (Figures 1C,D, 5A). During this time period, growth yields of the two mutants, which both highly produced the Mg^2+^ transporter MgtB (Snavely et al., 1991b; Figures 1D, 5A), were lower than that of the WT (Figures 1C, 5C), suggesting that cytoplasmic Mg^2+^ levels in the mutants could be higher than the WT levels. Together, these results suggest that the ΔLD and *mgtM*(UAG) strains induce MgtC and MgtB in a dysregulated manner under conditions in which low cytoplasmic Mg^2+^ stress does not exist.

The F_1_F_0_ ATP synthase inhibitory protein MgtC (Lee et al., 2013) contributes to growth of WT *Salmonella* in low Mg^2+^ (Blanc-Potard and Groisman, 1997; Rang et al., 2007). MgtC directly acts on the F_1_F_0_ ATP synthase and reduces ATP levels, while MgtB has no effect (Figure 2A; Lee et al., 2013). Abnormally high ATP levels cause a growth defect of the *mgtC* mutant in low Mg^2+^ (Pontes et al., 2015). By contrast, in the ΔLD strain, MgtC and MgtB reduced ATP at levels lower than the WT levels in a process that did not require F_1_F_0_ ATP synthase (Figure 2), which appeared to impair bacterial growth (Figures 1E, 2C and Supplementary Figure S5).

The ΔLD and *mgtM*(UAG) strains produced ∼4- and ∼2-fold higher levels of MgtC than the WT, respectively, whereas MgtB levels were similar among the strains (Figure 5B). However, growth of the ΔLD strain was more impaired than the *mgtM*(UAG) strain (Figures 2A, 5). These findings suggest that overproduction of MgtC might impair growth of *Salmonella* in whatever environment it finds itself. However, this scenario is unlikely because of the following observations. The regulatory peptide MgtR specified by the *mgtCBR* operon binds to and promotes MgtC degradation (Alix and Blanc-Potard, 2008). Although the Δ*mgtR* strain produced ∼4-fold higher levels of MgtC than the WT (Supplementary Figure S6A), the mutant grew in a manner similar to the WT strain in 10 μM Mg^2+^ (Supplementary Figure S6B). The Δ*mgtR* strain also overproduced MgtC only in the slow-growth phase (Supplementary Figure S6A), resembling the temporal production of MgtC in the WT strain (Figure 1D). Moreover, even if MgtC was produced in a dysregulated manner, its inhibitory effect was not observed unless MgtB is produced in a dysregulated manner. We engineered the Δ*mgtC* and ΔLD Δ*mgtC* strains to express the *mgtC* ORF from the plasmid-linked *lac* promoter. When placed in medium with 10 μM Mg^2+^ and the same concentrations of IPTG, the two strains produced comparable levels of MgtC after 1 h (Supplementary Figure S7A). However, the Δ*mgtC* and ΔLD Δ*mgtC* strains produced MgtB in manners similar to the WT and ΔLD strains, respectively (Supplementary Figure S7A). Furthermore, ATP reduction and growth defect resembling the phenotypes of ΔLD strain were observed in the ΔLD Δ*mgtC* strain but not in the Δ*mgtC* strain (Supplementary Figures S7B,C). Together, all of these lines of evidence reinforce that dysregulated expression of MgtC and MgtB leads to ATP reduction, which impairs *Salmonella* growth.

The molecular basis of how the dysregulated induction of MgtC and MgtB reduces ATP levels independently of the F_1_F_0_ ATP synthase is currently unclear. MgtC affects membrane potential in a manner not associated with the ability to inhibit the F_1_F_0_ ATP synthase, suggesting that MgtC also affects ATP levels by acting on a protein(s) other than the F_1_F_0_ ATP synthase (Lee et al., 2013). The Δ*mgtC* strain harbors a hyperpolarized membrane, whereas *mgtC* overexpression causes membrane depolarization in WT *Salmonella* (Lee et al., 2013). Interestingly, *mgtB* overexpression in WT *Salmonella* also depolarizes a membrane (Lee et al., 2013). Moreover, the MgtB transporter is one of proteins that are crosslinked to the MgtC protein (Lee et al., 2013), suggesting direct interaction between these two proteins. Based on these lines of evidence, we hypothesize that when produced in a dysregulated manner, MgtC and MgtB together act on the protein(s) that affects membrane potential to cause membrane depolarization, which in turn reduces ATP to abnormal levels.

To survive inside the macrophage phagosome, *Salmonella* induces *mgtC* expression (Blanc-Potard and Groisman, 1997). MgtC acts on the F_1_F_0_ ATP synthase to reduce intracellular ATP, otherwise, ATP levels increase upon acidic pH inside the phagosome (Lee et al., 2013). The coupling/uncoupling of transcription of the *mgtCBR* mRNA leader with the translation of *mgtM* and *mgtP* responds to distinct intracellular cues (i.e., an increase in ATP and a decrease in proline, respectively), inducing a conformation of the leader that allows transcription elongation into the *mgtC*-coding region (Lee and Groisman, 2012a, b; Lee et al., 2014). These events occur independently but additively to each other and enable *Salmonella* to achieve *mgtC* expression at levels that ensure its survival inside macrophages (Lee et al., 2014). In addition to these findings, our data suggest that preventing dysregulated induction of MgtC by the *mgtCBR* mRNA leader is another important determinant for intramacrophage survival of *Salmonella*. During infection of macrophages, WT *Salmonella* induced detectable MgtC at 6 h but not at 1 h (Figure 4A), which is in good agreement with the previous detection of MgtC at 20 h but no detection at 5 h post-infection (Yeom et al., 2018). By contrast, the ΔLD and *mgtM*(UAG) strains induced MgtC at the early time point and showed greatly attenuated survival inside macrophages (Figures 4, 5).

The role of *cis-*acting regulatory RNAs in preventing dysregulated gene expression also impacts the pathogenesis of another bacterial species. In *Yersinia pseudotuberculosis*, the *yscW*–*lcrF* operon specifies the transcriptional regulator LcrF (Skurnik and Toivanen, 1992). The intergenic region of the *yscW*–*lcrF* mRNA functions as a thermosensor, forming a secondary structure that permits translation of *lcrF* at host body temperature (Bohme et al., 2012). Mutations that destabilize this structure enable LcrF production at lower temperatures, resulting in LcrF-dependent expression of virulence genes and attenuated bacterial virulence in mice (Bohme et al., 2012). However, the molecular basis underlying the virulence attenuation remains unknown. We determined that the Mg^2+^ transporter MgtB, which is not normally associated with the virulence phenotype (Figure 4C; Blanc-Potard and Groisman, 1997), impairs intramacrophage survival of the ΔLD and *mgtM*(UAG) strains in the presence of MgtC (Figures 4C, 5F). Moreover, given that the ΔLD strain exhibited the defective phenotype in the absence of normal F_1_F_0_ ATP synthase function (Figure 4D), these results suggest that the unusual ATP reduction by dysregulated expression of MgtC and MgtB could impair growth of *Salmonella* inside host cells.

## Data Availability Statement

All datasets generated for this study are included in the article/Supplementary Material.

## Author Contributions

MP, HK, DN, and DS conceived the research. MP, HK, and DN performed the research. MP, HK, DN, D-HK, and DS analyzed the data. MP, HK, D-HK, and DS wrote the manuscript.

## Conflict of Interest

The authors declare that the research was conducted in the absence of any commercial or financial relationships that could be construed as a potential conflict of interest.
