# Supplementary material for: The mgtCBR mRNA Leader Secures Growth of Salmonella in Both Host and Non-host Environments
Source: Front Microbiol. 2019 Dec 6;10:2831. doi: 10.3389/fmicb.2019.02831 (PMC6908480; doi:10.3389/fmicb.2019.02831)
Supplement: Supplementary file 1 [file Data_Sheet_1.PDF]

Supplementary Material

The *mgtCBR* mRNA leader secures growth of *Salmonella* in both host and non-host environments

Myungseo Park, Hyunkeun Kim, Daesil Nam, Dae-Hyuk Kweon*, and Dongwoo Shin*

*Corresponding authors:

Dae-Hyuk Kweon. Email: dhkweon@skku.edu

Dongwoo Shin. Email: shind@skku.edu

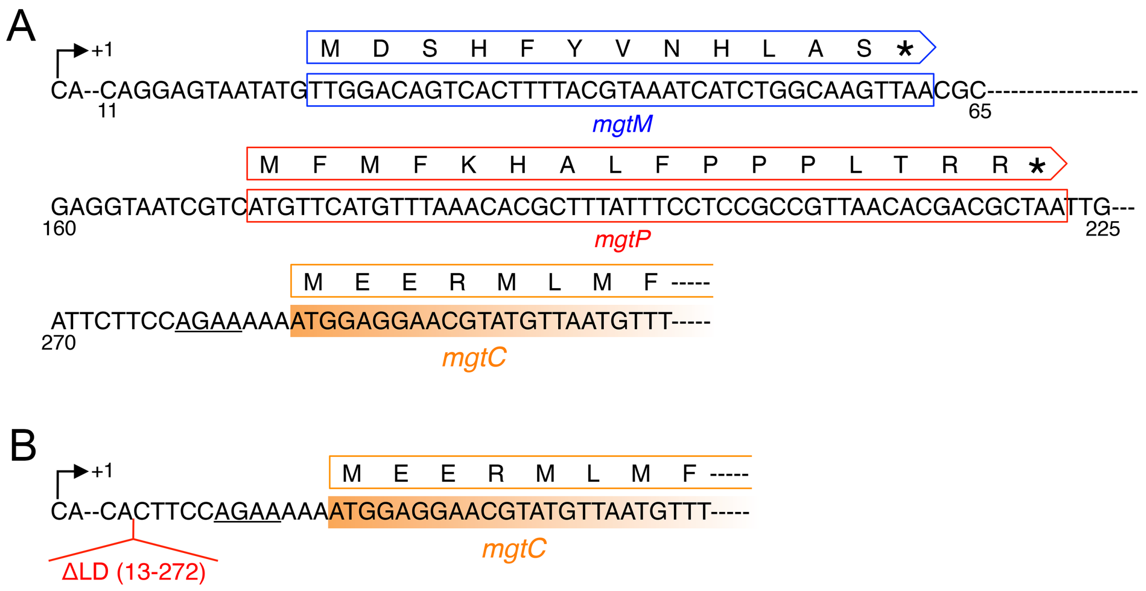

Supplementary Figure S1. Nucleotide sequences corresponding to the *mgtCBR* mRNA leader regions in wild-type (A) and ΔLD (B) strains. The transcription initiation sites (+1) are indicated as arrows, and the *mgtC* ribosome-binding sites are underlined. Numbering here is based on the transcription initiation site.

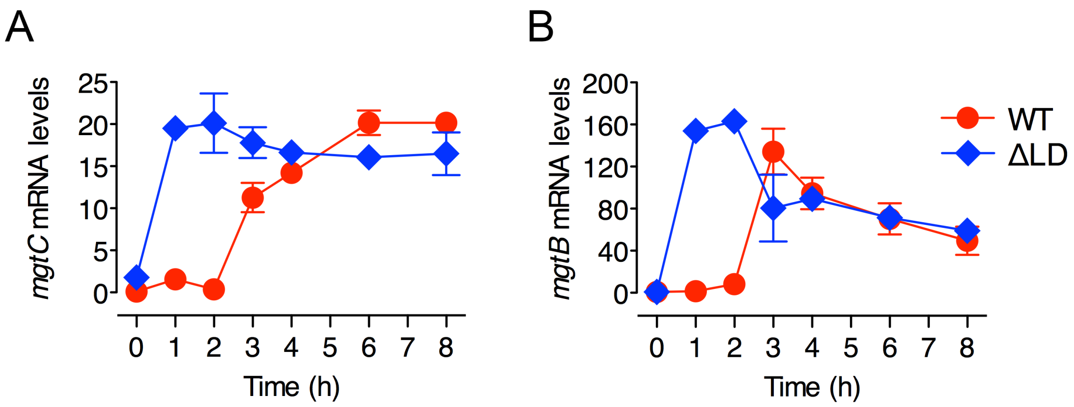

Supplementary Figure S2. The ΔLD strain ectopically produces the *mgtC* and *mgtB* mRNAs in low Mg^{2+}. qRT-PCR analysis determined mRNA levels corresponding to the *mgtC*- (A) and *mgtB*-coding (B) regions in wild-type (WT, 14028s) and ΔLD (DN557) strains. Bacteria were grown in N-minimal medium with 10 μM Mg^{2+} at pH 7.5, and bacterial RNA was isolated at the indicated time points. Means and standard deviations from three independent experiments are shown.

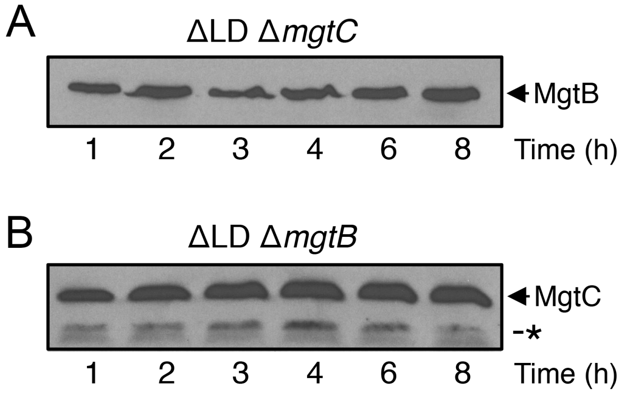

Supplementary Figure S3. Immunoblot analysis of MgtC and MgtB was conducted on crude extracts prepared from ΔLD Δ*mgtC* (HK111) (A) and ΔLD Δ*mgtB* (DN581) (B) strains. Bacteria were grown in N-minimal medium with 10 μM Mg^{2+} at pH 7.5 and harvested at the indicated time points. The band indicated with an asterisk (*) corresponds to a protein displaying cross-reactivity against the anti-MgtC antibody.

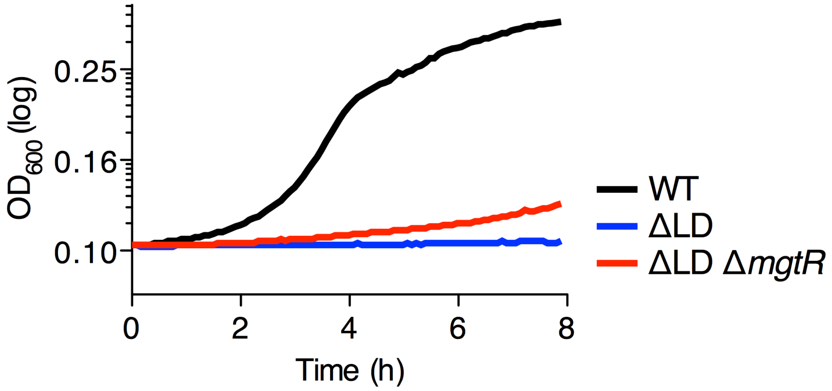

Supplementary Figure S4. Deletion of the *mgtR* gene does not recover growth of ΔLD *Salmonella*. Growth curves of wild-type (14028s), ΔLD (DN557), and ΔLD Δ*mgtR* (DN582) strains were obtained. Bacteria were grown in N-minimal medium with 10 μM Mg^{2+} at pH 7.5, and OD_{600} values were determined every 5 min up to 8 h using a plate reader. Data are representative of two independent experiments.

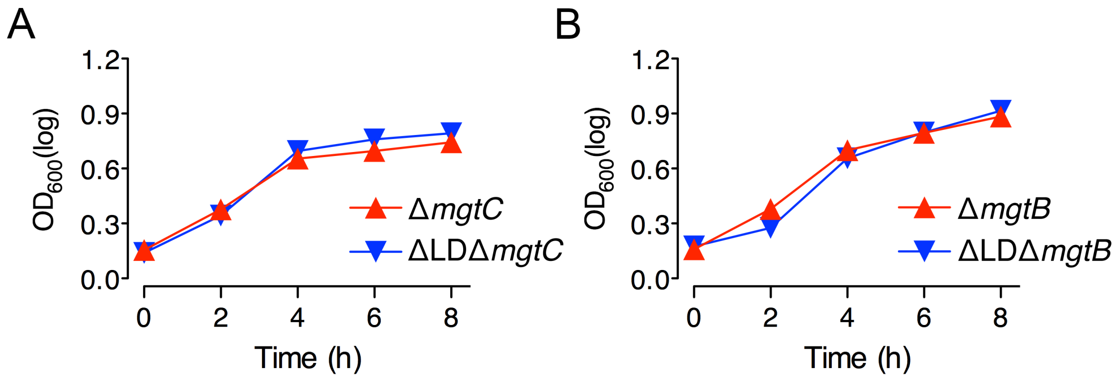

Supplementary Figure S5. Growth curves of *Salmonella* strains, Δ*mgtC* (EN397), ΔLD Δ*mgtC* (HK111), Δ*mgtB* (EN481), and ΔLD Δ*mgtB* (DN581). Bacteria were grown in N-minimal medium with 10 μM Mg^{2+} at pH 7.5, and OD_{600} values were determined at the indicated time points. Data are representative of three independent experiments.

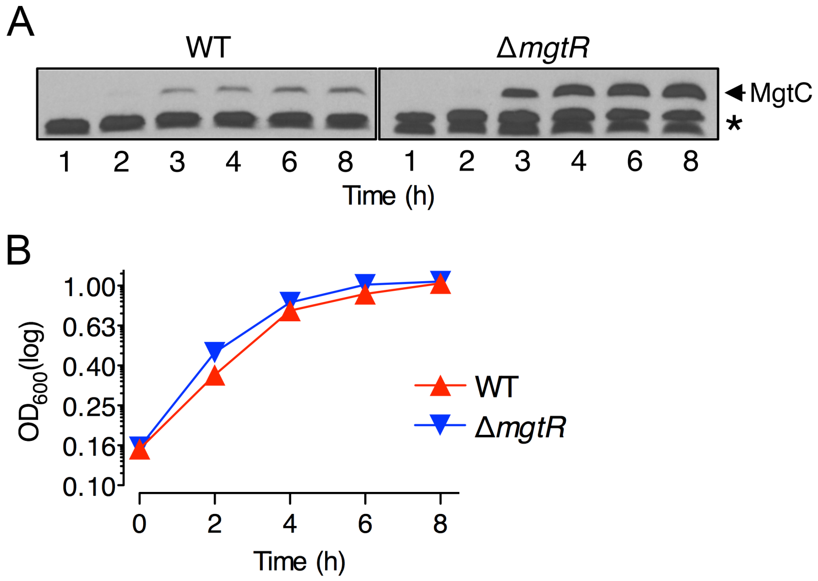

Supplementary Figure S6. The effect of *mgtR* on MgtC production and on bacterial growth was examined. Bacteria were grown in N-minimal medium with 10 μM Mg^{2+} at pH 7.5. (A) Immunoblot analysis of MgtC was conducted on crude extracts prepared from wild-type (WT, 14028s) and Δ*mgtR* (DN552) strains. MgtC levels were determined at the indicated time points. The band indicated with an asterisk (*) corresponds to a protein displaying cross-reactivity against the anti-MgtC antibody and serves as an internal loading control. (B) Growth curves of wild-type and Δ*mgtR* strains. OD_{600} values were determined at the indicated time points. Data are representative of two independent experiments.

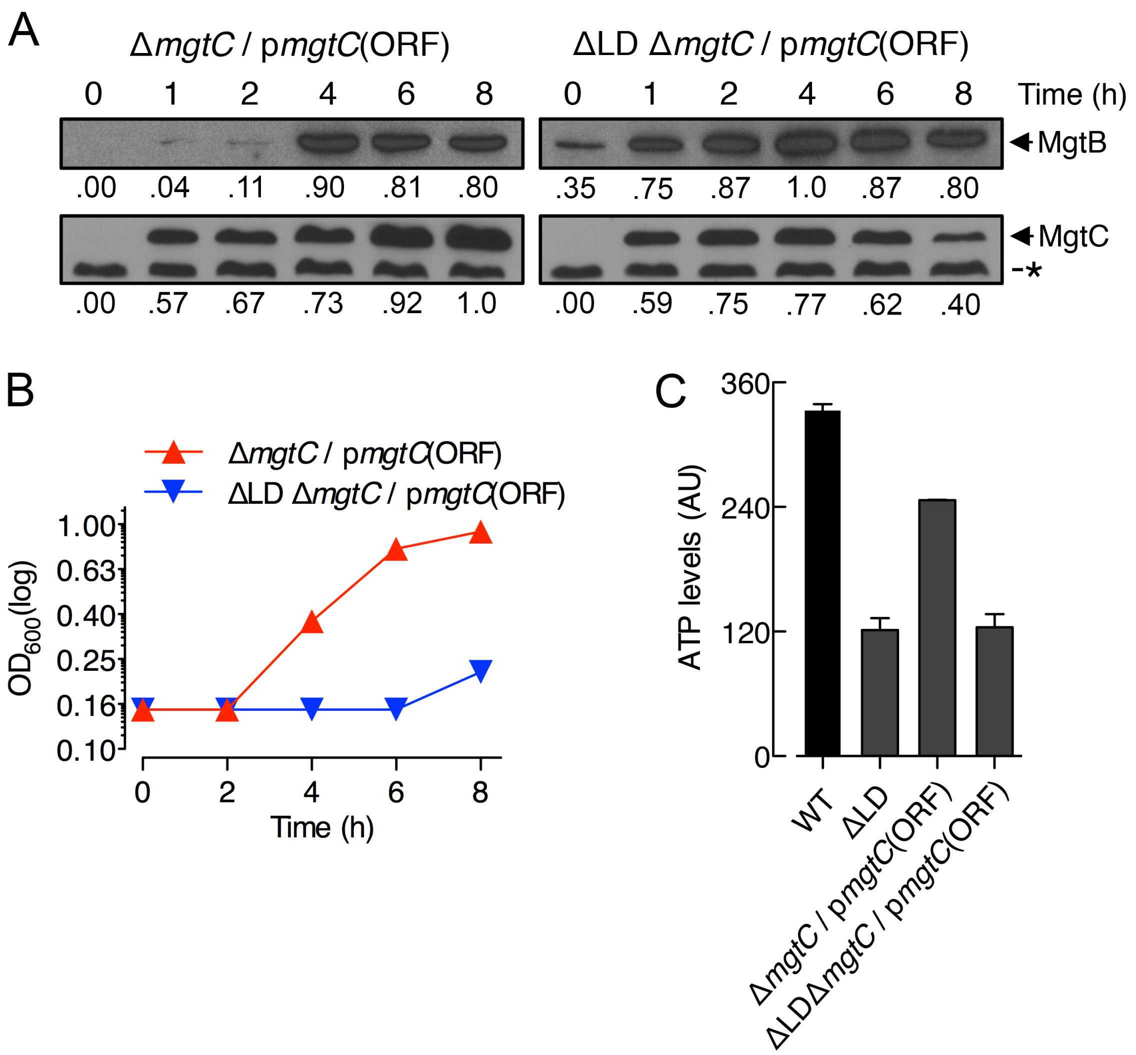

Supplementary Figure S7. Heterologous expression of *mgtC* impairs *Salmonella* growth when MgtB is ectopically produced. Bacteria were grown in N-minimal medium with 10 μM Mg^{2+} at pH 7.5. (A) Immunoblot analysis of MgtC and MgtB was conducted on crude extracts prepared from Δ*mgtC* (EN397) and ΔLD Δ*mgtC* (HK111) strains, each of which harbors p*mgtC*(ORF) plasmid. MgtC and MgtB levels were determined at the indicated time points. The band indicated with an asterisk (*) corresponds to a protein displaying cross-reactivity against the anti-MgtC antibody and serves as an internal loading control. Numbers below the blots correspond to relative levels of MgtC and MgtB at a given time point. (B) Growth curves of Δ*mgtC*/p*mgtC*(ORF) and ΔLD Δ*mgtC*/p*mgtC*(ORF) strains. OD_{600} values were determined at the indicated time points.

Data are representative of two independent experiments. (C) ATP levels of wild-type (WT, 14028s), ΔLD (DN557), Δ*mgtC*/p*mgtC*(ORF), and ΔLD Δ*mgtC*/p*mgtC*(ORF) strains grown for 4 h. Data depicted in arbitrary units (AU) are means and standard deviations from three independent experiments. IPTG was used at 0.5 mM to induce expression of the *mgtC* ORF from the plasmid.

Supplementary Table S1. Primers used in this study.

Primers	Sequences (5' to 3')
Del701	TCATCTGGCAAGTTAACGCACGCTATTCCTGCGCTGCTTGTTAAGACCCACTTTCACATTTAAG
Del702	TATTGGGATGACAATCGTCACAAGGGGAGATTGCTGCCCACTAAGCACTTGTCTCCTGTTTAC
Del703	ACAGAGCTATCGCCGGTATTAAGCAGG
Del704	GAATAAAAAACTATGCATGAATTTGCGCTTATTATAGTCA
Del705	GCAAATTCATGCATAGTTTTTTATTCAATTGCAACATTAC
Del706	CATACGTTGACGCCATTGTCTTTCCGC
Del708	TTACTCACTATTTTGAGTGGGTTTT
Del711	ATATGCAGGAAACACTACACCTTAATTTTGGGGATTCATCTGTAGGCTGGAGCTGCTTCG
Del712	TATCGGGTGAGCGATTCATCTGGGCGATCCTCAAACATTACATATGAATATCCTCCTTAG
Del713	CGTGTGCTAAATATAGCACGTACTTATTCTTCCAGAAAAATGTAGGCTGGAGCTGCTTCG
Del714	ATCATTCCCTCCTTATACGCCTGGCGTAATGTTGCAATTGCATATGAATATCCTCCTTAG
Del715	CTTTGGCCAGTGGTTTTAATAATGTTTGAGGATCGCCCAGTGTAGGCTGGAGCTGCTTCG
Del716	AAATTTTGTCCAACCCTCTTTTTTGCATGGCGTCACCTCGCATATGAATATCCTCCTTAG
Del717	TGGCGCCGGCTGTAATTAACAACAAAGGGTAAAAGGCATCTGTAGGCTGGAGCTGCTTCG
Del718	CTCCAGTTTGTTTCAGTTAAAACGTAGTAGTGTTGGTAAACATATGAATATCCTCCTTAG
Ex301	ATTTCACACAGAATTCTGGAGGAACGTATGTTAATGTTTCCTTATA
Ex302	GCTTGGCTGCAGTTATTGACTATCAATGCTCCAGTGAAT
Q-mgtC-F	TTGTCTCTGGGATTGGCTTTCT
Q-mgtC-R	CAGCCCGCGCACATTC
Q-mgtB-F	TCGAAGCGGAAGCTTTTCAT

Q-mgtB-R	TCATTGCGCCCATACACTTT
Q-gyrB-F	ACGCGTCTGTTGACCTTCTTC
Q-gyrB-R	CTGTTCCTGCTTACCTTTCTTCAC
